# Prediction of Nonalcoholic Fatty Liver Disease Via a Novel Panel of Serum Adipokines

**DOI:** 10.1097/MD.0000000000002630

**Published:** 2016-02-08

**Authors:** Raika Jamali, Abbas Arj, Mohsen Razavizade, Mohammad Hossein Aarabi

**Affiliations:** From the Research Development Center, Sina Hospital/Digestive Disease Research Institute, Non-Alcoholic Fatty Liver Disease Center, Tehran University of Medical Sciences, Tehran (RJ); Department of Internal Medicine, Shahid Beheshti Hospital (AA, MR); and Research Center for Biochemistry and Nutrition in Metabolic Disease, Faculty of Medicine, Kashan University of Medical Sciences, Kashan, Iran (MHA).

## Abstract

Considering limitations of liver biopsy for diagnosis of nonalcoholic liver disease (NAFLD), biomarkers’ panels were proposed. The aims of this study were to establish models based on serum adipokines for discriminating NAFLD from healthy individuals and nonalcoholic steatohepatitis (NASH) from simple steatosis.

This case-control study was conducted in patients with persistent elevated serum aminotransferase levels and fatty liver on ultrasound. Individuals with evidence of alcohol consumption, hepatotoxic medication, viral hepatitis, and known liver disease were excluded. Liver biopsy was performed in the remaining patients to distinguish NAFLD/NASH. Histologic findings were interpreted using “nonalcoholic fatty liver activity score.” Control group consisted of healthy volunteers with normal physical examination, liver function tests, and liver ultrasound. Binary logistic regression analysis was applied to ascertain the effects of independent variables on the likelihood that participants have NAFLD/NASH.

Decreased serum adiponectin and elevated serum visfatin, IL-6, TNF-a were associated with an increased likelihood of exhibiting NAFLD. NAFLD discriminant score was developed as the following: [(−0.298 × adiponectin) + (0.022 × TNF-a) + (1.021 × Log visfatin) + (0.709 × Log IL-6) + 1.154]. In NAFLD discriminant score, 86.4% of original grouped cases were correctly classified. Discriminant score threshold value of (−0.29) yielded a sensitivity and specificity of 91% and 83% respectively, for discriminating NAFLD from healthy controls. Decreased serum adiponectin and elevated serum visfatin, IL-8, TNF-a were correlated with an increased probability of NASH. NASH discriminant score was proposed as the following: [(−0.091 × adiponectin) + (0.044 × TNF-a) + (1.017 × Log visfatin) + (0.028 × Log IL-8) − 1.787] In NASH model, 84% of original cases were correctly classified. Discriminant score threshold value of (−0.22) yielded a sensitivity and specificity of 90% and 66% respectively, for separating NASH from simple steatosis.

New discriminant scores were introduced for differentiating NAFLD/NASH patients with a high accuracy. If verified by future studies, application of suggested models for screening of NAFLD/NASH seems reasonable.

## INTRODUCTION

Nonalcoholic liver disease (NAFLD) is a common cause of cirrhosis and liver-related morbidity and mortality. It is already considered the hepatic manifestation of metabolic syndrome. The prevalence of NAFLD is increasing worldwide due to the alterations in lifestyle and the epidemic of obesity and insulin resistance (IR). Accumulation of visceral adipose tissue (VAT) and the development of IR seem to be pivotal to the steatohepatitis (NASH) and concomitant fibrosis.^1^ Early diagnosis and appropriate management of NAFLD might have a great impact on public health through minimizing complications. Ischemic heart disease, cerebrovascular accident, chronic kidney disease, diabetes mellitus, and liver-related morbidities (comprising liver transplantation and hepatocellular carcinoma) are among the NAFLD complications.^2^

Liver biopsy remains the gold standard for precise diagnosis of NAFLD in patients. However, application of this procedure is limited in clinical setting due to invasiveness, cost, concerns about sampling error, and potential serious complications. Therefore, unraveling an alternative to accurately diagnose patients with NAFLD is crucial. In this regard, various panels of serum biomarkers have been proposed in different cohorts of NAFLD populations.^2^ A combination of clinical data and serum markers that may be related to the pathogenesis of NAFLD was applied to design these panels. There is still paucity of evidence on accuracy of these models. Adipokines that are hormones secreted by VAT are implicated in development of obesity and pathogenesis of IR.^1^ Recently, there is great enthusiasm in studying their potential role in the pathogenesis of NAFLD. Notably, previous studies showed the relationship of mentioned adipokines with NAFLD/NASH.^3–9^ In this research, we aim to evaluate the association between some important adipokines, in particular adiponectin, visfatin, resistin, hepcidin, as well as inflammatory cytokines of IL-6, IL-8, and TNF-α with NAFLD.

The aims of this study were: to assess the correlation of clinical parameters, mentioned serum adipokines, and inflammatory cytokines with the presence of NAFLD/NASH; to establish a model to discriminate NAFLD from healthy subjects and NASH from simple liver steatosis.

## METHODS

### Ethical Considerations

The study was conducted according to ethical standards for human experimentation (Helsinki Declaration). The ethics committee of the hospital approved the study protocol (No: 8861). The purpose of the study was explained to the participants and they were enrolled in the study after filling out the written consent.

### Patient and Control Enrolment Protocol

This case-control study was conducted in a general university hospital between 2012 and 2014. The patient group consisted of subjects with persistently elevated serum aminotransferase levels and well-defined criteria of fatty liver in ultrasound examination. They were consecutively admitted to the hepatology clinic (Phase 1).^10,11^ The upper normal limit for serum aminotransferases was considered 40 units per liter.^12^ Individuals with evidence of alcohol consumption, hepatotoxic medication, viral hepatitis, and known liver disease were excluded (Phase 2).^13^ The remaining patients in Phase 2 were presumed to have NAFLD. Liver biopsy was performed in this group to document NAFLD before final enrolment (Phase 3). Control group consisted of age and sex-matched healthy volunteers who accompanied the patients to the gastroenterology clinic. The gastroenterologist examined the controls to evaluate their health status. They were enrolled into the study if there was no evidence of fatty liver at their liver ultrasound examination and abnormal routine laboratory investigation including liver function tests. A statistician who was unaware of participants’ data used the block-matching method (with control to case ratio of 1:1) for the matching of the controls.

### Liver Ultrasonography

Fatty infiltration in liver scatters the beam of ultrasound more than normal liver tissue; therefore, the fatty liver appears hyperechogenic in ultrasound examination. The comparison of echogenicity is required with internal structures known to be void of fat, such as the kidney. In this study, the radiologist obtained the sagittal view of liver right lobe and right kidney for the evaluation of fatty liver.^11^

### Sample Size Calculation

The sample size was determined by a statistical power analysis. Considering the mean prevalence of NAFLD (*P* = 28%) according to the previous studies, α=0.05, and *d* = 0.12, the sample size was calculated as 54 in NAFLD group.^11^

### Laboratory Assays

The fasting serum samples were obtained to assess fasting blood glucose, insulin, lipid profile, liver function tests (LFT), adiponectin, visfatin, hepcidin, resistin, IL-6, IL-8, and TNF-α concentrations. All of the measurements were performed by enzyme-linked immunosorbent assay (ELISA) according to the manufacturer's instructions. To minimize the laboratory errors, the same operator performed the whole assay, from the beginning to the end, and room temperature, air humidity, and incubator temperature were controlled. All the measurements were performed in duplicate. The intra-assay coefficient variations were less than 12%. The laboratory investigators were blinded to the case or control status of participants. The following ELISA kits were used in this study: human adiponectin and visfatin ELISA kits (Production numbers: AG-45A-0001 and AG-45A-0006 respectively; ADIPOGEN Inc, South Korea), resistin (human resistin ELISA kit, Biovendor, Czech Republic), hepcidin (Lot: RN- 24429; DEMEDITEC GmbH, Kiel-Wellsee, Germany), IL-6 (Lot: 233737; Bendered Systems GmbH, Vienna, Austria), IL-8 (Lot: ab46032; IL-8 human ELISA kit, Abcam, USA), and TNF-α (Lot: ab46087; TNF-α human ELISA kit, Abcam). Other serum assays including fasting blood glucose, insulin, lipid profile (triglyceride, total cholesterol, high-density lipoprotein, low-density lipoprotein), and LFT (alanine aminotransferase, aspartate aminotransferase, alkaline phosphatase, gamma glutamyl transpeptidase) were performed based on methodology of previous studies.^11–14^

### Liver Histology

Percutaneous liver biopsy was performed in patients with NAFLD using true cut needle (G14). The acceptable liver biopsy sample size was considered sample with 10-mm length or containing at least 5 portal tracts after fixation in formaldehyde (10%) and staining. Hematoxyline-eosin stain was used for the evaluation of necroinflammation. A pathologist who was blinded to the patient data interpreted the slides. The degree of liver steatosis, lobular inflammation, and fibrosis were defined based on “nonalcoholic fatty liver activity score” (NAS). The patients with score equal to 5 or higher were presumed to have NASH. Those with scores equal to 2 or lower were considered to have simple liver steatosis.^15^

### Statistical Analysis

Continuous variables were reported as mean (± standard deviation) and categorical variables were shown as count (percent). For univariate analysis, *t* test and *χ*^2^ tests were applied to assess differences between groups, where appropriate. Binary logistic regression analysis using standard model was applied to ascertain the effects of independent variables (including adipokines, inflammatory cytokines, metabolic profile, liver function tests, and clinical data) on the likelihood that participants have NAFLD/NASH. Standardized correlation coefficients (beta) with 95% confidence intervals (CI) were calculated. The biomarkers that were independently associated with NAFLD/NASH were selected for discriminant function analysis (DFA). Kolmogorov–Smirnov test was used for the assessment of normality distribution of biomarkers. In order to transform the distribution of non-normal variables to normal, the logarithm of non-normal distributed biomarkers was calculated and entered for DFA. This analysis determined the weight of the biomarkers that discriminated between study groups by defining the standardized canonical discriminant function coefficients. Finally, equations were developed based on DFA to calculate the discriminant scores, by weighted combination of discriminating biomarkers. Receiver operating characteristic (ROC) analysis was performed to determine the threshold values of discriminant scores for differentiating NAFLD from control group and NASH from simple liver steatosis. The best cut-off values were determined in order that the sum of sensitivity and specificity was the highest. All statistical analyses were performed by SPSS, version 17 (SPSS Inc, Chicago, IL). The probability of difference between variables was considered statistically significant if 2-sided *P* value was less than 0.05.

## RESULTS

Seventy participants suspected of having NAFLD were evaluated from September 2012 to September 2014 (Step 1). Reasons for leaving out were patient unwillingness to participate in the study (n = 8), normalization of ALT during the lead-in phase (n = 6), autoimmune hepatitis (n = 1), and viral hepatitis (n = 1) (Step 2). Finally, 54 biopsy proven NAFLD patients were included in the study (Step 3). The comparisons of clinico-demographic and laboratory data between patients and controls are demonstrated in Table 1.

**TABLE 1:**
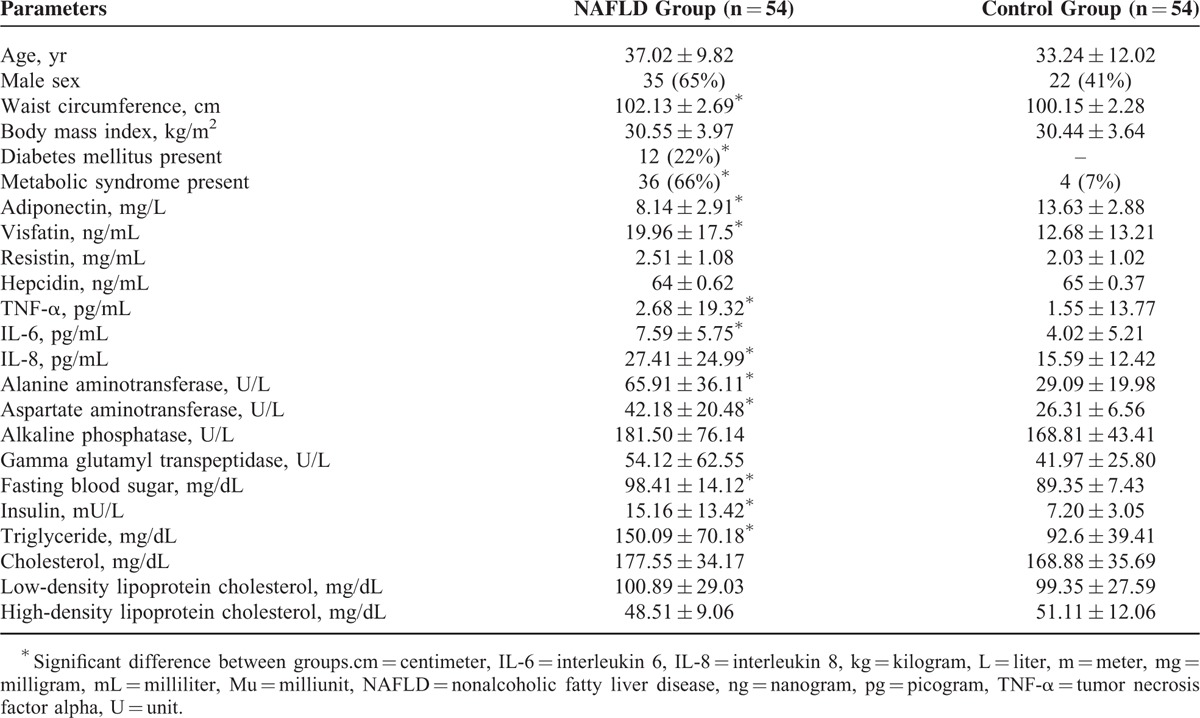
Comparison of Clinico-Demograhic Characteristics, Metabolic Profile, Serum Adipokines, Inflammatory Cytokines, and Liver Function Tests in Study Groups

**FIGURE 1:**
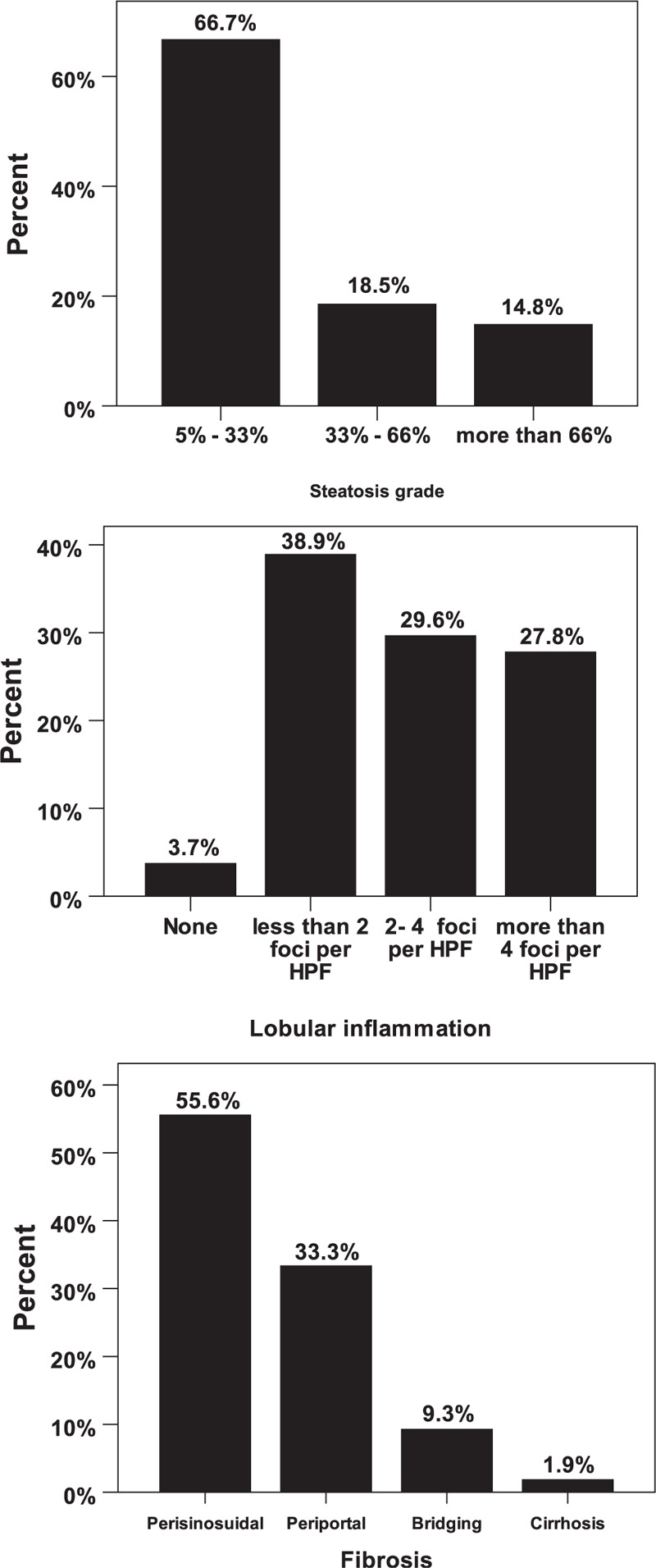
The frequency (percent) of histologic findings in nonalcoholic fatty liver group. The degree of steatosis (top), lobular inflammation degree based on foci of lobular inflammation in high power field of microscopic view (middle), and fibrosis degree (bottom).

The mean (±SD) NAS score was 4.87 (± 1.71) in the NAFLD group. The percent of the patients with an NAS score of 5 or more (those with steatohepatitis) was 31.7%. The percent frequency of patients with steatosis grade of less than 33% was 66.7%; meanwhile, the frequency of those with steatosis grade of more than 33% was 33.3% (Figure 1). The frequencies of the patients with lobular inflammation of less than 2 foci and more than 2 foci per high power field (HPF) were 42.6% and 57.4% respectively (Figure 1). The most common type of fibrosis was perisinusoidal fibrosis (Figure 1).

In binary logistic regression, decreased serum adiponectin and elevated serum visfatin, IL-6, TNF-α were independently associated with an increased likelihood of NAFLD presence (Table 2). The best threshold values of the above-mentioned biomarkers for differentiating nonalcoholic fatty liver disease patients from healthy subjects according to ROC analysis are provided in Table 3. Using standardized canonical discriminant function coefficients derived from DFA, the equation for calculation of discriminant score (for separating NAFLD from controls) was constructed as follows:

**TABLE 2:**
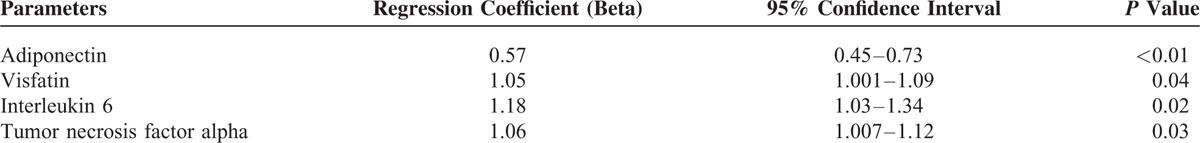
Independent Predictors of Nonalcoholic Fatty Liver Disease

**TABLE 3:**
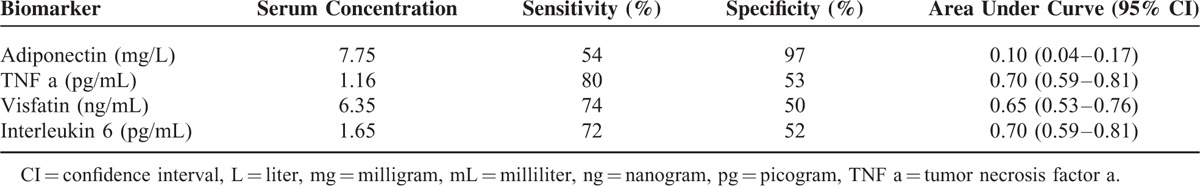
Best Threshold Values of Biomarkers for Differentiating Nonalcoholic Fatty Liver Disease Patients From Healthy Subjects According to Receiver Operating Characteristic (ROC) Analysis

NAFLD discriminant score: [(−0.298 × adiponectin) + (0.022 × TNF-α) + (1.021 × Log visfatin) + (0.709 × Log IL-6) + 1.154].

**FIGURE 2:**
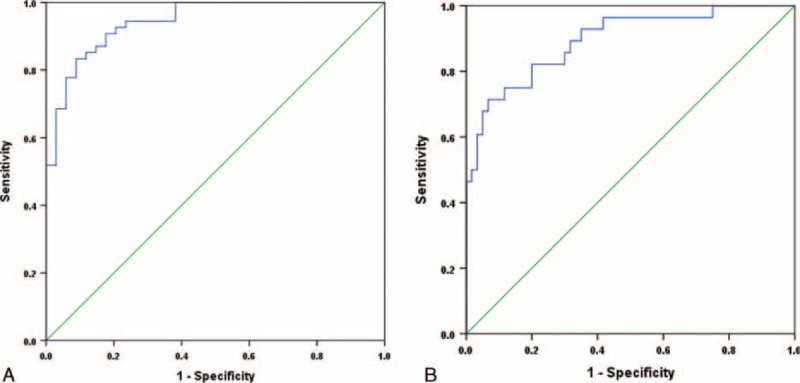
Receiver operating characteristic (ROC) curves for the proposed models. A, ROC curve for various cut-off levels of proposed discrimination score in differentiating between nonalcoholic fatty liver disease patients and controls (area under curve: 94%, 95% CI: 90%–98%). B, ROC curve for various cut-off levels of proposed discrimination score in differentiating between nonalcoholic steatohepatitis and simple liver steatosis patients (area under curve: 90%, 95% CI: 82%–97%).

The classification result of DFA showed that 86.4% of original grouped cases were correctly classified. ROC analysis depicted that the discrimination score threshold value of (−0.29) yielded a sensitivity and specificity of 91% and 83% respectively, for separating NAFLD from healthy subjects (Figure 2A).

In binary logistic regression analysis, decreased serum adiponectin and elevated serum visfatin, IL-8, TNF-α were associated with an increased likelihood of NASH presence (Table 4). Applying a similar method, with regard to the calculation of discriminant score (for separating NASH from simple steatosis cases) the following equation was developed:

**TABLE 4:**
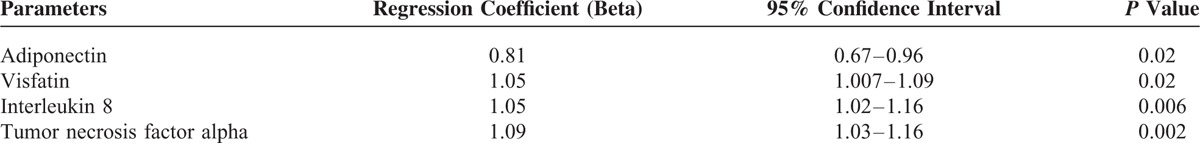
Independent Predictors of Nonalcoholic Steatohepatitis

NASH discriminant score: [(−0.091 × adiponectin) + (0.044 × TNF-α) + (1.017 × Log visfatin) + (0.028 × Log IL-8) − 1.787].

DFA showed that 84% of original and 77% of cross-validated grouped cases were correctly classified. ROC analysis demonstrated that the discrimination score threshold value of (−0.22) yielded a sensitivity and specificity of 90% and 66% respectively, for separating NASH from simple steatosis (Figure 2B).

## DISCUSSION

The current research revealed the reverse association of serum adiponectin and positive association of visfatin, IL-6, and TNF-α with the presence of NAFLD. It also demonstrated the increased probability of NASH presence with decreased serum adiponectin and elevated levels of circulating visfatin, IL-8, and TNF-α. New formulas were developed to discriminate NAFLD from control group and NASH from simple liver steatosis. To the best of our knowledge, the proposed models in this research exhibited higher accuracy than the previously developed models for discrimination of NAFLD/NASH (Table 5).^16–27^

**TABLE 5:**
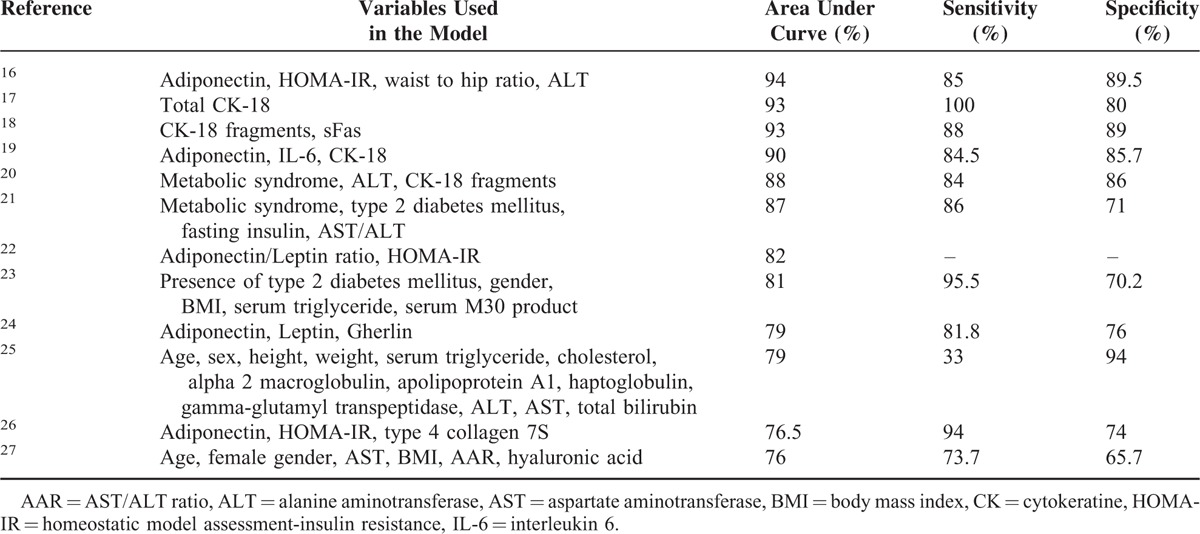
Characteristics of Developed Models for Prediction of Nonalcoholic Fatty Liver Disease

To define noninvasive methods for precise detection of NAFLD/NASH, the current research assessed a wide range of clinical as well as serologic parameters. Considering the importance of adipokines in NAFLD pathophysiology, we investigated the association between a panel of serum adipokines and presence of NAFLD/NASH.

In the present investigation, hypoadiponectinemia was associated with NAFLD/NASH. This finding is in concordance with previous experiments.^3–9^ Adiponectin might serve as predictor of NAFLD in obese children.^3^ Decreased adiponectin levels were the primary hint in the course of NAFLD, even before rise of proinflammatory cytokines.^28^ Also, adiponectin played an important role in the progression of simple liver steatosis to NASH.^29^ Low serum adiponectin levels were evident before the development of overt diabetes and obesity.^30^ Decrease in circulating adiponectin levels was associated with reduced liver insulin sensitivity and increased liver fat content. Moreover, hypoadiponectinemia was suggested as a part of metabolic disturbance that was characterized by accumulation of VAT.^31^ Previous studies recommended application of serum adiponectin as a diagnostic measure and a therapeutic target for NAFLD.^32^ In the present research, serum visfatin levels were significantly associated with NAFLD/NASH that is in line with previous studies.^33–36^ Notably, elevation in circulating visfatin was parallel to the pancreatic beta cell dysfunction in diabetics.^34^ Likewise, serum visfatin was correlated with systemic IR and development of metabolic syndrome.^35^ On the other hand, decreased visfatin levels in VAT were correlated with NAFLD.^4^ Circulating visfatin was significantly decreased in the end stage liver disease presumably due to decreased hepatic production.^36^ In this study, serum TNF-α levels were related to the presence of NAFLD/NASH that is comparable to the findings by other researchers.^4,37^ TNF-α might have a role in liver cell inflammation and fibrosis by development of IR in NASH.^5^ Based on this evidence, some experts suggested that anti TNF-α medication might be a potential progress for NASH treatment.^37^ Also, circulating IL-6 levels was higher in NAFLD group than the controls in the present investigation that is parallel to the outcome of previous observations.^6,7,38^ Similarly, serum IL-6 levels were associated with the severity of hepatocyte inflammation, stage of fibrosis, and systemic IR in patients with NASH.^38^ It was demonstrated that insulin sensitivity is increased in obese mice by application of IL-6 antibodies. Chronic treatment with IL-6 also inhibited activation of insulin receptor in liver.^39^ These observations emphasized the significance of IL-6 in hepatic IR in NAFLD. In accordance with the literature, serum IL-8 was associated with NASH in this research.^7^ With regard to hepcidin, the circulatory level was not different between NAFLD and control group in our study. Meanwhile, a previous study demonstrated higher hepcidin levels in NAFLD subjects; nevertheless, the research found no correlation between hepcidin and histologic findings.^8^ It was demonstrated that body iron stores in NAFLD regulated hepcidin.^40^ Therefore, it seems reasonable to adjust for patients iron storage when evaluating hepcidin levels in NAFLD patients. Another finding of current observation was that serum resistin levels were higher in NAFLD than healthy subjects; however, the difference was not statistically significant. This finding is in accordance with a previous study that showed the correlation of resistin with the presence of steatosis and necroinflammation in NAFLD.^41^ Meanwhile, another research demonstrated the association of low serum resistin levels with excessive fat accumulation in liver.^42^

According to the results, the correlations of some adipokines were higher than the other studied variables with the presence of NAFLD. Although LFT and metabolic indices are routinely used for diagnosis and monitoring of NAFLD, it seems that some adipokines might be used for this purpose as well. In this regard, identifying the role of VAT-derived secretory proteins (including proinflammatory cytokines and polypeptide hormones) in the pathogenesis of NAFLD is of great value. Designing therapeutic approaches by the modulation of associated adipokine might have great impact on disease burden. Noteworthy, aminotransferase levels fluctuate in the course of NAFLD; therefore, the results of this study need to be evaluated in further prospective trials.^43^

There is currently no defined “normal range” for serum adipokines. Moreover, adipokine levels might also fluctuate over-time according to the metabolic environment. These concerns might impact the accuracy of the proposed models in this study.

Several models have been proposed based on clinical and laboratory parameters for screening of NAFLD/NASH. Assessment of variables in the introduced models in the current project is simple and safe. However, cost-effectiveness of such panels needs to be further studied. The classification results of DFA in these models documented that a high percentage of original grouped cases were correctly classified. ROC curves also showed a great accuracy with respect to the high area under curve (AUC) of both developed models. The characteristics of previous developed models are summarized in Table 4 for further comparison. The head-to-head evaluation of the proposed panel in this study with the previously developed models is a potential future research direction. One weakness of the study is that the prediction of the study's accuracy is only measured in the internal cohort of patient samples that generated the model equations. It would greatly benefit the strength of the study, if the accuracy could be measured in an “external” or independent cohort of patients.

The development of NASH biomarkers can be theoretically achieved via 2 different strategies. The first strategy can be defined as “knowledge-based” (deductive method based on the current knowledge of NASH pathophysiology), while the second one is more “unbiased” (inductive strategy). The “knowledge-based” approach relies on a direct understanding of the pathophysiological processes that underlie the development of NASH as well as the evolution of its sequelae. It may consist of biochemical assays aiming to assess attractive novel candidate markers informed by the biology of disease process. For instance, the understanding of the role played by hepatocyte apoptosis and insulin resistance in the pathobiology of liver injury has enabled the development of promising biomarkers of NASH, such as caspase-cleaved cytokeratin 18 fragments or numerous different adipokines. On the other hand, the “unbiased” approach involves the use of modern techniques including proteomics, metabolomics, and bioinformatics that have allowed unbiased investigations of numerous putative markers that may be informative with regard to the various stages of NAFLD, including overt NASH and its sequelae.^44^ In order to construct our panel, we selected the biomarkers with the high evidence for their role in NAFLD (ie, adiponectin, visfatin, resistin, hepcidin, IL-6, IL-8, and TNF-alpha) using a “knowledge-based” approach.

The relationships between few serum adipokines with NAFLD/NASH were evaluated in individual reports previously.^3–9^ In this research, several key adipokines together with metabolic profiles and LFT were evaluated, providing an advantage to previous studies. To improve the power of study, the cases were selected from a cohort of biopsy-proven NAFLD/NASH subjects. In this research, we used the NAS system for histologic description of NAFLD/NASH. This model is a validated scoring system for NAFLD that interpret the spectrum of disease with an excellent reliability and degree of agreement.^15^

The individuals with known liver disease were excluded from this study and the mean age of participants was 37. These facts indicate the early stage of NAFLD in the studied cohort. Consequently, the current results could not be generalized to all NAFLD patients. Well-controlled studies are recommended for the validation of the new proposed formula for NAFLD/NASH determination in patients with advanced disease including cirrhotic and pretransplantation. Liver histology is now considered the gold standard method for detection of NAFLD/NASH; however, there are controversies on type 2 error. Since only small amount of liver (1/50,000) is evaluated in liver biopsy, sampling error is a concern. The distribution of necroinflammation throughout the liver is not homogenous especially in the early stage of NAFLD. It was proposed that scoring systems would potentially characterize a more reliable sign of global liver damage severity than is obtained by the liver biopsy.^45,46^ Justification of the proposed biomarker panels with regard to follow-up and response to treatment in NAFLD patients is recommended.

## Conclusion

Our suggested models for predicting NAFLD and NASH based on serum adipokines show promising accuracy to detect patients with NAFLD/NASH.

## Summary

The association between a panel of serum biomarkers (including adipokines, LFT, and metabolic profile) and clinical data with presence of NAFLD/NASH was evaluated in this study. We introduced new models for discriminating NAFLD from healthy subjects as well as patients with NASH from those with simple liver steatosis based on a panel of serum markers. The models demonstrated great accuracy with regard to their significant AUC. The defined threshold values based on these discriminant scores showed decent sensitivity and specificity.
